# Impact of complete dentures treatment on Oral health-related quality of Life (OHRQoL) in edentulous patients: A descriptive case series study

**DOI:** 10.12669/pjms.40.9.9402

**Published:** 2024-10

**Authors:** Shameen Tariq, Ahmad Shoaib, Farooq Ahmad Chaudhary, Syed Rashid Habib, Muhammad Qasim Javed

**Affiliations:** 1 Shameen Tariq, FCPS Prosthodontist, Rawalpindi Institute of Cardiology, Rawalpindi, Pakistan; 2 Ahmad Shoaib, FCPS, Assistant Professor, Prosthodontics, School of Dentistry, Shaheed Zulfiqar Ali Bhutto Medical University, Islamabad, Pakistan; 3 Farooq Ahmad Chaudhary, PhD, Associate Professor of Community Dentistry, School of Dentistry, Shaheed Zulfiqar Ali Bhutto Medical University, Islamabad, Pakistan; 4 Syed Rashid Habib, FCPS, Department of Prosthetic Dental Sciences, College of Dentistry, King Saud University, PO. Box 60169, Riyadh, 11545, Saudi Arabia; 5 Muhammad Qasim Javed, FCPS, Associate Professor, Department of Conservative Dental Sciences, College of Dentistry, Qassim University, Buraidah, PO box 1162, Qassim 51452, Saudi Arabia

**Keywords:** Complete denture, Edentulism, GOHAI, Oral health-related quality of life

## Abstract

**Objectives:**

To evaluate the changes in oral health-related quality of life [OHRQoL] before and three months after complete denture oral rehabilitation using the Geriatric oral health assessment index [GOHAI] in Pakistani elderly edentulous patients.

**Methods:**

In this descriptive case series study a total of 80 edentulous patients were recruited from Islamic International Dental Hospital, Islamabad, Pakistan from November 2021 to April 2022. The OHRQoL was evaluated twice by using the GOHAI- Urdu version. First, before getting the complete denture, and subsequently after three months of using a prosthesis. The data were analysed using SPSS-23. Pre and post-comparison were done by t-test. Moreover, the ANOVA test was utilized to assess the socio-demographic variables-GOHAI score and association.

**Results:**

The mean GOHAI scores increased significantly from 22.9 (SD = 3.01) at baseline to 28.1 (SD = 5.59) after three months (P = 0.001), reflecting an overall increase of 5.2 points. At baseline, the ability of the patient to swallow comfortably was found to have the highest mean GOHAI score of 3.02 (SD= 1.24) and after three months of complete denture insertion, the same questionnaire item further improved with the highest mean GOHAI score of 3.43 (SD=1.05). The socio-demographic characteristics-based comparison of mean pre and post-insertion GOHAI scores suggested a statistically significant difference (p=0.001).

**Conclusion:**

The OHRQoL in elderly edentulous patients improved after complete denture delivery, and a significant improvement was noted in most GOHAI domains.

## INTRODUCTION

Aging is a natural biological process that brings about an array of social, psychological, and physical changes in a person’s life.^1^ Care for the elderly is crucial, given the extent of these health issues, to ensure they can continue living healthily with a better quality of life (QoL). Common oral health issues in the elderly include loss of teeth, oral and pharyngeal cancer, orofacial pain, mucosal oral lesions, xerostomia, and edentulism.^2^-^4^

Edentulism is an irreversible oral condition in which no teeth are present in entire dental arches seen commonly in the elderly population. Studies have shown that it is more prevalent in people with lower socioeconomic status and can cause impairment in oral functions and psycho-social disability.^5^,^6^ Complete denture (CD) treatment is the preferred option for treating edentulism in developing countries because of its easy maintenance, appealing aesthetics, and low cost.^7^ The clinicians generally base the outcome of CD treatment on their own clinical assessments of parameters such as its retention, support, esthetics, occlusion, etc., and often neglect the patient’s view and perception. However, in the recent decade, clinicians have started to recognize the significance of patients’ view, and perspective regarding their oral health and treatments with its impacts on their life.^8^-^10^ The use of different OHRQoL measures such as Oral Health Impact Profile [OHIP]; the Oral Impacts on Daily Performance [OIDP]; Geriatric/General Oral Health Assessment Index [GOHAI], assess the oral health and treatment outcomes from the perspective of patients through functional and psycho-social constructs of health.^8^,^11^-^14^ To have an overall assessment of a CD treatment outcome it needs to assess the perspective of patients on this treatment and its impact on their OHRQoL.^15^

The OHRQoL can be improved if the CD is properly working and enhances the patients’ oral functionality, appearance, and social life, comfort. However, it can have a negative impact on OHRQoL if the CD is unstable or uncomfortable. Therefore, it is vital to evaluate the OHRQoL in CD patients periodically. Despite the growing body of research on OHRQoL, there is a notable scarcity of studies assessing changes in OHRQoL following CD treatment in the Pakistani elderly population. Moreover, the use of OHRQoL questionnaires to assess quality of life in this context is rare. This research aims to fill this gap by evaluating changes in OHRQoL before and three months after CD oral rehabilitation using the Geriatric Oral Health Assessment Index (GOHAI) - Urdu version, in Pakistani elderly edentulous patients. This study is unique in its focus on a specific, under-researched population and its use of a culturally adapted OHRQoL measure, providing valuable insights into the impact of CD treatment in a developing country context.

The aim of the study was evaluate the changes in oral health-related quality of life [OHRQoL] before and three months after complete denture oral rehabilitation using the Geriatric oral health assessment index [GOHAI] in Pakistani elderly edentulous patients

## METHODS

The edentulous patients for this descriptive case series study were recruited from, Islamic International Dental Hospital, Pakistan from November 2021 to April 2022. The edentulous patients aged between 60-85 years, getting CD treatment for the first time in their life were recruited using a non-probability consecutive sampling technique. The suitability of the cases was judged through history, clinical, and radiographic examination. Those patients who had any temporomandibular joint disorders, highly resorbed residual ridge, were mentally handicapped, had uncontrolled diabetes, and history of radiation therapy to the head and neck area were excluded.

**Fig.1:**
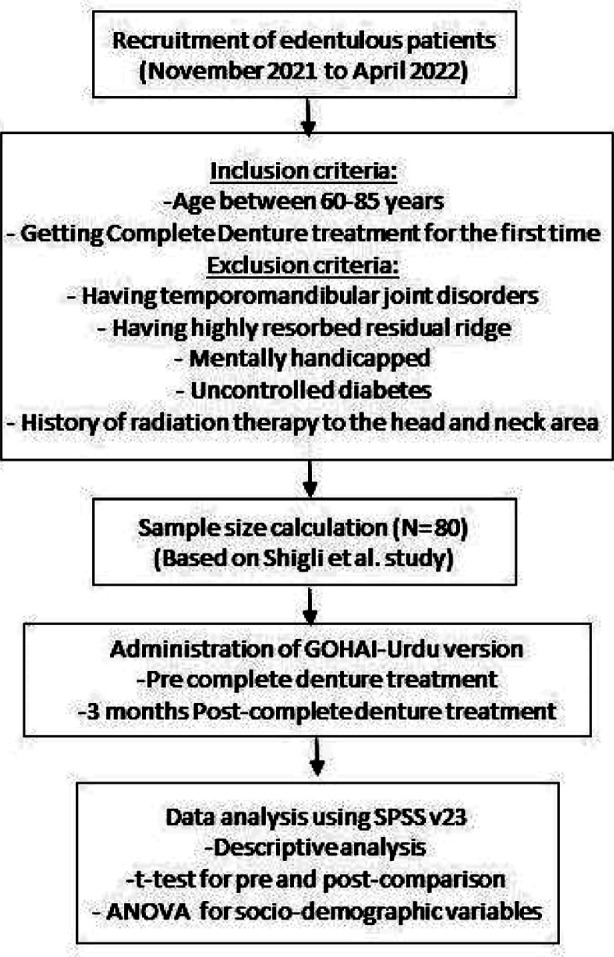
Flowchart of study methodology.

A sample size of 80 patients was calculated using results (μ = 2.71, Sd=1.46) of the study by Shigli et al., at a 95% confidence level and 5% error.^11^ Ethical approval was acquired from the ethics board of Riphah International University (Ref. No. IIDC/IRC/20/09/015, dated: September 5, 2020). Additionally, written consent was obtained from research participants. The GOHAI-Urdu version was administered to assess the OHRQoL of the participants, first, before providing the CD, and later after three months of CD insertion.^16^ GOHAI is a 12-items self-assessing questionnaire, assessing three assumed functions Pain/discomfort, physical and psychosocial functions) of OHRQoL.^13^ However, in this study, the 12th question was omitted, which asked about dental sensitivity as all the participants included in this study were edentulous. The participants responded on a 5-point Likert scale [1= always to 5= never). The cumulative score was determined by summing up all items’ scores. The total score of 11 items GOHAI for this study ranged from 11 to 55, with higher scores showing better OHRQoL.^13^ The flow chart of the study methodology is given in Fig.1.

The data were analysed using SPSS-23 with a 5% significant level. Descriptive analysis was carried out to describe the sample and the measure. Pre and post-comparison were done by t-test. Moreover, the ANOVA test was utilized to assess the socio-demographic variables-GOHAI score and association.

## RESULTS

A total of 80 edentulous patients having 70.4 years mean age were recruited for this study. The majority were male (73.8%) and had educational levels between matric to inter (44.4%) (Table-I). The change in the mean GOHAI score among baseline (22.9, SD= 3.01) and after three months (28.1, SD= 5.59) was 5.2. At baseline, the ability to swallow comfortably had the highest mean GOHAI score of 3.02 (SD= 1.24) and after three months of CD insertion, the same item further improved with the highest mean GOHAI score of 3.43 (SD=1.05) followed by being pleased with the looks (3.00, SD= 1.11) (Table-II).

**Table-I:** Description of the sample (N= 80).

Characteristics	Number (%)
***Age*** *Mean(SD)*	70.4 (6.62)
60-65	32 (40.0)
66-70	6 (7.5)
71-75	12 (15.0)
76-80	27 (33.7)
81-85	3 (3.75)
***Gender***	
Male	59 (73.8)
Female	21 (26.2)
***Education level***	
Uneducated	8 (10.0)
Matric	18 (22.5)
Inter	38 (47.5)
Graduate and Above	16 (20.0)
***Living***	
Urban	41 (50.6)
Rural	39 (48.1)

**Table-II:** GOHAI mean score changes in pre-insertion and post-insertion of complete denture.

Questions	Pre-insertion Mean [SD]	Post- insertion Mean [SD]	Score- change (Post–Pre)	Percentage change (%)
“1. How often did you limit the kinds or amounts of food you eat because of problems with your teeth or denture?”	1.78±0.74	2.70±1.06	0.92	34.07
“2. How often did you have trouble biting or chewing any kinds of food, such as firm meat or apples?”	1.95±0.76	2.93±1.10	0.98	33.44
“3. How often were you able to swallow comfortably?”	3.02±1.24	3.43±1.05	0.31	9.93
“4. How often have your teeth or dentures prevented you from speaking the way you wanted?”	1.58±0.83	2.18±1.33	0.6	27.52
“5. How often were you able to eat anything without feeling discomfort?”	2.27±1.00	2.76±0.88	0.49	17.75
“6. How often did you limit contacts with people because of the condition of your teeth or denture?”	1.38±0.58	2.00±1.21	0.62	31.0
“7. How often were you pleased or happy with the looks of your teeth, gums or dentures?”	2.45±1.12	3.00±1.11	0.55	22.4
“8. How often were you worried or concerned about the problems with your teeth, gums or dentures?”	2.36±0.90	2.68±0.80	0.32	11.94
“9. How often did you use medication to relieve pain or discomfort from around your mouth?”	1.46±0.69	1.41±0.80	-0.05	3.54
“10. How often did you feel nervous or self-conscious because of problems with your teeth, gums or dentures?”	2.13±0.72	2.95±0.84	0.82	27.7
“11. How often did you feel uncomfortable eating in front of people because of problems with your teeth or dentures?”	1.65±0.61	2.91±1.31	1.26	43.2
Total	22.9 (3.01)	28.1 (5.59)	5.2	18.50
P-value	0.001			

The socio-demographic characteristics-based comparison of mean pre and post-insertion GOHAI scores suggested a statistically significant difference (p=0.001). Female patients (8.90, SD=5.15) showed greater improvement in OHRQoL than male patients (3.83, SD=5.02), similarly, patients aged between 60-65 years (8.40, SD=4.85) showed the highest improvement in OHRQoL compared all other age groups. The improvement in OHRQoL is also greater in patients with high education (8.31, SD=5.38) and those living in urban areas (7.04, SD=5.87) (Table-III).

**Table-III:** Mean comparison of pre and post-insertion GOHAI scores between the socio-demographic characteristics.

	GOHAI score Mean (Sd)			

	Post insertion	Pre insertion	Paired mean difference	P-value
***Gender***				
Male	23.2 (3.13)	27.1 (5.28)	3.83 (5.02)	0.001
Female	22.0 (2.50)	31.0 (5.54)	8.90 (5.15)	
***Age Mean(SD)***				
60-65	22.8 (2.10)	31.2 (4.95)	8.40 (4.85)	
66-70	25.5 (2.73)	27.0 (2.19)	1.50 (0.54)	
71-75	21.5 (2.71)	24.5 (2.71)	3.00 (3.04)	0.001
76-80	23.0 (3.84)	26.1 (6.06)	3.11 (6.12)	
81-85	25.0 (0.00)	30.0 (0.00)	5.00 (0.00)	
***Education Level***				
Uneducated	26.6 (3.96)	27.1 (3.90)	0.50 (0.53)	0.001
Matric	22.8 (2.40)	25.0 (4.27)	2.22 (4.08)	
Inter	22.4 (3.00)	28.6 (6.23)	6.21 (5.56)	
Graduate and Above	22.5 (1.89)	30.8 (4.51)	8.31 (5.38)	
***Residence***				
Urban	22.6 (2.76)	29.6 (5.45)	7.04 (5.87)	0.001
Rural	23.3 (3.25)	26.4 (5.31)	3.17 (4.33)	

## DISCUSSION

This study assessed the physical and psychosocial aspects and traits that may affect the overall success of the rehabilitation treatment in edentulous patients using a psychometric measure GOHAI. Their opinion and feedback about the quality of the prostheses worn by them would provide a guideline for the dentist to improve and emphasize the requirement for considering the patient’s perceptions and concerns.

The quality of life (QoL) for edentulous individuals is significantly diminished due to masticatory impairments, resulting in a reduced intake of essential calories and nutrients necessary for maintaining health.^17^ Furthermore, research indicates a higher prevalence of hyposalivation among older adults who are edentulous or have a reduced number of teeth.^18^ The oral health-related quality of life (OHRQoL) also deteriorates due to the functional and psychosocial impacts of edentulism. People without teeth often experience low self-esteem and psychological issues, leading them to avoid social interactions and activities, and sometimes resulting in complete social isolation.^5^,^19^

In this study, the OHRQoL was assessed twice including pre-insertion and three months post-insertion of CD using the GOHAI-Urdu version. Studies have shown inconsistent results regarding the changes in OHRQoL immediately after insertion of a CD and recommended time for assessing OHRQoL after 6-12 weeks post insertion.^15^,^20^,^21^ In this study, the GOHAI score increased significantly after three months post-insertion as compared to pre-insertion as indicated by an 18.5% improvement in summary score. This result is in line with the findings of Sivakumar and colleagues, Shigli & Hebbal, and Ellis et al.,^9^,^11^,^15^ but in contrast with the studies by Veyrune et al., and Goiato et al.^22^, in which no difference was observed in GOHAI score between baseline assessment and after six weeks.^23^,^24^ Furthermore, some studies have shown a significant reduction in impairment level of OHRQoL 6 months and 12 months post insertion of complete dentures.^25^,^26^

A study conducted on the elderly population in France to assess OHRQoL following the receipt of new complete prostheses found that the GOHAI score did not show a statistically significant improvement after six weeks. However, there was a statistically significant improvement in the GOHAI score at the twelve-week mark after receiving complete dentures.^23^ Similarly, research on the elderly population in India reported comparable findings. The mean GOHAI score (25.08±1.18) aligned with the results of other studies conducted within Indian populations.^9^

In this study, the OHRQoL improved more in females compared to males post-insertion. Some studies have shown that males lose teeth faster and experience edentulism more than females.^26^ That is plausibly linked with male parafunctional oral habits such as betel nuts and pan chewing, more tobacco use including smoking, and bad oral care. In this study, the sample of female patients was very low (26%) which might be one of the reasons for better improvement of OHRQoL in female patients. The level of gender influence on rehabilitation is greatly inspired by the psychosocial difference between men and women. Studies have shown that women are more socially disabled in middle and low-income countries before the start of the denture treatment, which can be attributed to their attitude and concerns towards oral impairment and rehabilitation.^26^,^27^

In this study, patients in the younger age group (60-65 years) demonstrated a greater improvement in OHRQoL compared to older age groups. These findings are consistent with prior research, which indicates that younger patients tend to report more significant improvements in OHRQoL following complete denture (CD) treatment. Oral health issues generally escalate with age, leading older patients to often adopt more accepting attitudes towards their oral health problems, viewing them as a natural part of the aging process.^7^, ^11^ The paired mean difference among the various domains of GOHAI indicated positive improvements, except for the frequency of medication used to relieve pain or discomfort in the mouth. This exception is likely due to the discomfort and pain that some patients experience when first using complete dentures (CD), as it typically takes time for them to adjust to wearing the new prostheses.

Overall, improvement in the OHRQoL was seen after CD treatment, however, the magnitude of the improvement varies with some studies showing greater improvement while others lower after CD rehabilitation, mostly depending upon variation and variability of the sample, period of evaluation, and proper fitting dentures.^25^,^28^,^29^

### Limitation of the Study:

The small sample size, and sample recruitment from a single urban-based hospital using a nonrandomized technique limited the generalization of findings to the whole population.

### Strength of the Study:

These findings are important in predicting the possible impact of CD treatment in terms of patient well-being and can apply to other under-developed populations with similar socioeconomic and demographic characteristics like the Pakistani population.

## CONCLUSION

The GOHAI-Urdu can evaluate the need for complete dentures and detect OHRQoL over time after treatment in Pakistani elderly patients. The findings of this study revealed a significant improvement in OHRQoL following CD treatment, as indicated by the significant increase in mean GOHAI scores. Specifically, the ability to swallow comfortably showed the highest improvement. These findings are important for underdeveloped countries like Pakistan where most edentulous patients belong to lower socio-economic strata and CD therapy is the most feasible and affordable treatment option for them.

### Future Recommendations:

Future research with multicenter design, and focusing on a larger sample size and longer follow-up period are important for generalization of the findings.

### Authors Contribution:

**ST**, **SA:** Conceived, designed and did the data collection and reviewed the manuscript and responsible and accountable for the accuracy or integrity of the work.

**FAC**, **MQJ:** Did statistical analysis, write up & editing of manuscript.

**SRH:** Write up, editing, review and final approval of manuscript.
